# A bottom-up initiated digital external quality assessment scheme for the state-of-the-art pathology in Sweden: reduced variability between pathology departments

**DOI:** 10.1007/s00428-025-04059-9

**Published:** 2025-02-28

**Authors:** Gunilla Rask, Helena Olofsson, Annette Bauer, Anna Bodén, Johannes van Brakel, Eugenia Colón-Cervantes, Anna Ehinger, Anikó Kovács, Åsa Rundgren-Sellei, Johan Hartman, Josefin Ågren, Eva Darai-Ramqvist, Charlotta Andersson, Christina Kåbjörn Gustafsson, Balazs Acs

**Affiliations:** 1 Department of Medical Biosciences/Pathology, Umeå University, Umeå, Sweden; 2 Department of Diagnostics and Intervention, Surgery, Umeå University, Umeå, Sweden; 3 Department of Clinical Pathology, Västerås Hospital, Västerås, Sweden; 4 Pathology & Cytology Dalarna, County Hospital Dalarna, Falun, Sweden; 5 Department of Clinical Pathology, Linköping University, Linköping, Sweden, and Department of Biomedical and Clinical Sciences, Linköping University, Linköping, Sweden; 6 Department of Pathology, Skåne University Hospital, Malmö, Sweden; 7 Department of Clinical and Surgical Pathology, Unilabs, Stockholm, Sweden; 8 Department of Genetics, Pathology and Molecular Diagnostics, Laboratory Medicine, Region Skane, Lund University, Lund, Sweden; 9 Division of Oncology, Department of Clinical Sciences Lund, Lund University Cancer Center, Skåne University Hospital Comprehensive Cancer Center, Lund, Sweden; 10 Department of Clinical Pathology, Sahlgrenska University Hospital, Gothenburg, Sweden; 11 Institute of Biomedicine, Sahlgrenska Academy, University of Gothenburg, Gothenburg, Sweden; 12 Department of Clinical Pathology and Cancer Diagnostics, Karolinska University Hospital, Stockholm, Sweden; 13 Department of Oncology-Pathology, Karolinska Institutet, Stockholm, Sweden; 14 Equalis AB, Uppsala, Sweden; 15 Expert Group on Pathology and Cytology, Equalis AB, Uppsala, Sweden; 16 Swedish Breast Pathology Expert Group, KVAST Bröst, Uppsala, Sweden

**Keywords:** Breast neoplasms, Tumour biomarker, Laboratory proficiency testing, Quality assurance

## Abstract

External quality assessment (EQA) schemes for pathology are essential, yet large/international programmes do not assess morphology-based biomarkers or address local/regional needs. This study outlines bottom-up initiated, flexible Swedish Digital Pathology EQA rounds for breast pathology, and presents results from the 2021 and 2023 rounds. Six breast carcinoma cases were selected for each EQA round by the Swedish Breast Pathology Expert Group (KVAST Breast). Whole tissue slides stained with HE, IHC, and ISH were anonymized, digitized, and uploaded to the digital EQA platform. Biomarkers were selected based on national registry data analysis and pathologist and clinician feedback. The 2021 round assessed Nottingham grade (NHG), oestrogen receptor (ER), progesterone receptor (PR), and human epidermal growth factor receptor 2 (HER2), while the 2023 round focused on NHG, HER2-low, and global Ki67. Twenty-seven pathology departments participated. From 2021 to 2023, the variability of NHG assessment on digital slides improved from moderate to substantial (kappa 0.50; 95% CI 0.45–0.55 to 0.64; 95% CI 0.60–0.68), with better agreement for NHG3 than NHG1. Participants showed substantial and excellent agreement in ER (kappa 1) and PR (0.75 (95% CI 0.69–0.82). We found similar agreement in distinguishing HER2 IHC 0 (0.78; 95% CI 0.72–0.82) and HER2 IHC 3 + (0.94; 95% CI 0.88–1.00) from other HER2 IHC scores. Participants showed substantial agreement in detecting Ki67 high and Ki67 low cases (kappa 0.65; 95% CI 0.60–0.71 and 0.69; 95% CI 0.64–0.74, respectively). This digital EQA identifies local issues and complements large international EQAs to address challenges in the rapidly changing biomarkers of cancer therapy.

## Introduction

Every pathologist is aware of the importance of quality assessment (QA) schemes today. As cancer treatment becomes ever more personalized, we as pathologists must provide more and more complex details about the prognostic and predictive biomarkers of malignant tumours. Since the results of the pathologist’s histological, immunohistochemical, and molecular analyses form the basis for therapy decisions, it is crucial that these results are not flawed by any preventable factor relating to specimen handling, analytical steps in tissue stainings and immunohistochemical/molecular assays, image processing, data handling or lack of competence in personnel responsible for interpretation.

Many Swedish laboratories participate in external quality assessment schemes (EQAs) provided by large organizations like NordiQC, UKNEQAS, CAP-PT/EQA, or QuIP. While these schemes are robust, they lack two important aspects. Firstly, they focus on immunohistochemistry (IHC) and/or genetics, not on the basic histomorphological assessment of haematoxylin–eosin (HE) slides. Secondly, laboratories choose which programmes to participate in, meaning they will not find things they are not looking for. Contrary to popular belief, Swedish authorities in general rely little on detailed legislature to regulate companies or civil services, and laboratories are not required to be accredited or participate in any particular EQA schemes.

### What is KVAST?

The Swedish Society of Pathology and The Swedish Society of Clinical Cytology, run on voluntary basis, organize in total 15 groups for every organ/organ system called KVAST groups (KVAlitets- och STandardiseringskommittén, Committee for Quality and Standardization, *kvast* meaning broom in Swedish). These groups are composed of volunteer practising pathologists and operate in an open and inclusive manner to represent the entire Swedish pathology community. Although any practicing pathologist in Sweden can apply and be a member of the KVAST group, in general, there tends to be a higher representation from larger hospitals and university hospitals. Each KVAST group organizes EQAs in their pathology subject every other year based on the suggestions from pathologists, oncologists, and surgeons as a bottom-up approach. Furthermore, the KVAST groups organize educational activities and provide recommendations for national guidelines in questions concerning pathology and cytology.

### What is Equalis?

Equalis is a Swedish non-for-profit organization providing EQAs for laboratories in chemistry, microbiology, pathology, haematology, etc., as well as radiology (including ultrasound and nuclear medicine), and point-of-care analyses. Equalis is co-owned by the Swedish Association of Local Authorities and Regions, together with the professional associations of medical doctors (The Swedish Society of Medicine, SLS) and biomedical scientists (Institute of Biomedical Laboratory Science, IBL). Equalis provides all the administrative scaffolding for the EQAs in pathology, including ownership and maintenance of the Equalis Online portal (Equalis.se) where participants view cases, submit answers, and read the final reports after each EQA round.

In this study, we aimed to present the latest rounds of the Digital Swedish Pathology EQA scheme (2021 and 2023) that focused on the most critical and problematic biomarkers (human epidermal growth factor receptor 2—HER2 low positivity, Ki67 and Histological grade (NHG)) in the clinical decision-making of breast cancer care.

## Methods

The KVAST group for breast pathology organized digital EQA rounds in 2021 and 2023 targeting all pathologists who sign out breast cases, not only breast pathologists. All EQA rounds within digital pathology at Equalis follow a standardized schedule. Three to six months before the round, the responsible KVAST group decides on a topic and selects cases. The cases are collected from the clinical practice of the KVAST members’ hospitals and may contain HE slides, IHC, and/or in situ hybridization (ISH). The digitized, anonymized slides are sent to Equalis with a list of questions and a corresponding list of possible answers for each question and case. For example, a question may be *which grade the tumour has* and the answers would be *Nottingham grade, 1*, *2*, or* 3*. The slides and corresponding questions/answers are uploaded by Equalis to the online portal. Log-in details to the Equalis Online portal are sent to the members of the KVAST group, and each member then reviews the cases and submits their answers to the questions independently. In cases with disagreement within the KVAST group, there are three options: (a) resolution by consensus, (b) replacing the case with another if the disagreement is due to technical problems, or (c) allowing both answers to be correct. This last option is unique to the pathology EQAs at Equalis and reflects the acceptance that pathologists sometimes need to make a more clinically oriented decision rather than providing a purely analytical answer. If this option is chosen one of the answers is labelled “Expected answer” usually meaning what the majority of the KVAST group prefers, and one is labelled “Accepted answer”. For example, a tumour deemed HER2 1 + by most of the group members may have an accepted answer of 2 + . Pathologists who are not sub-specialized in breast pathology will frequently have a lower threshold for calling 2 + and ordering ISH in borderline cases. This reduces the risk of missing a HER2-amplified tumour. The selected cases and the expected/accepted answers are finalized 2 months before the round is opened, to allow time for quality checks.

The participating laboratories may submit individual responses from multiple assessors, later grouped by experience (resident, specialist < 5 years, or specialist > 5 years). Each participating lab is also requested to give an answer called “department answer”, wherein the evaluation should represent the department answer according to its regular routines. Only this department answer is used to compare results between laboratories. The purpose of these separate answers (individual and department) is to encourage young pathologists, or pathologists who are unfamiliar with the field but interested in learning, to participate in the EQA without affecting the score of the department. Rounds are open for 4 weeks. A more detailed report is sent to the KVAST group, allowing the group to analyze the results and get an overview of all laboratories (anonymized). The KVAST group writes a report for Equalis commenting on the result of the nation’s pathology laboratories as a whole, and with suggestions for future EQAs. A month after the round closing date, all the results and the comments from the KVAST group are published on the Equalis Online portal for all participants to read. Participants can see their own answers compared to the answers of the other participants, but who provided which answer is confidential. In October each year, The Swedish Society of Pathology and The Swedish Society of Clinical Cytology hosts a meeting where all EQA rounds within digital pathology from Equalis since the last meeting are presented by each KVAST group. All the participating laboratories in the EQA rounds are invited to this meeting. The analytic phase of breast cancer biomarkers is assessed in the international EQA programmes. Therefore, the Swedish breast digital EQA program is focused on the interpretation and scoring aspects.

### The 2021 round

The 2021 EQA round consisted of five cases of invasive breast carcinomas, which the participants were asked to score for glandular formation, nuclear atypia, and mitotic frequency, as well as providing a final Nottingham grade. Grade was included because according to national guidelines women with ER + , HER2 negative carcinoma are offered chemotherapy when grade 3 but recommended endocrine therapy only when grade 1. Thus, monitoring the variability in grading across the country is of great importance. Each case was also scored for ER, PR, HER2 IHC, and HER2 SISH, as well as given a designation as either HER2-positive or HER2-negative. As this was before the “HER2-low” era, no distinction was made between 0 and 1–2 + , non-amplified in the final HER2 status. One of the goals of the round was to draw attention to the five groups of HER2 SISH that were included in the 2018 WHO book, but not widely used in Sweden: Group 1—HER2/CEP17 ≥ 2 and ≥ 4 copies HER2/cell (amplified); Group 2—HER2/CEP17 ≥ 2 and < 4 copies HER2/cell (non-amplified, with comment); Group 3—HER2/CEP17 < 2 and ≥ 6 copies HER2/cell (amplified); Group 4—HER2/CEP17 < 2 and 4- < 6 copies HER2/cell (non-amplified, with comment); Group 5—HER2/CEP17 < 2 and < 4 copies HER2/cell (non-amplified). Table 1 summarizes the features of the cases chosen for the 2021 round.

**Table 1:** Expected answers (accepted answers) for cases in the 2021 round

	NHG	ER	PR	HER2 IHC	HER2 SISH	HER2 status
Case 1	3	51–100%	51–100%	2 +	Group 5	Non-amplified
Case 2	2	51–100%	51–100%	2 + (1 +)	Group 2 (5)	Non-amplified
Case 3	2	51–100%	0 (1–9%)	2 + (1 +)	Group 4 (5)	Non-amplified
Case 4	1 (2)	51–100%	51–100%	2 +	Group 5	Non-amplified
Case 5	3	0 (1–9%)	10–20% (21–50%)	3 +	Group 1	Amplified

*NHG* Nottingham histologic grade, *ER* oestrogen receptor, *PR* progesterone receptor, *HER2* human epidermal growth factor receptor 2, *SISH* silver in situ hybridization. Each cell shows the expected answer and (acceptable answer), if applicable. SISH groups according to WHO: group 1—HER2/CEP17 ≥ 2 and ≥ 4 copies HER2/cell; group 2—HER2/CEP17 ≥ 2 and < 4 copies HER2/cell; group 4—HER2/CEP17 < 2 and 4–6 copies HER2/cell; group 5—HER2/CEP17 < 2 and < 4 copies HER2/cell

### The 2023 round

The 2023 EQA round entailed six cases of invasive breast carcinomas where the participants were asked to evaluate Nottingham grade, HER2 immunohistochemistry, and global Ki67. ER and PR were omitted since there were no indications from the 2021 round or registry data that the scoring of these biomarkers was problematic. The grade was included for the same reason as previously. HER2 scoring was purposely focused on IHC only. It was known that antibody–drug conjugates approved for so called “HER2-low” disease would in all probability be approved in Sweden during 2024, and that all pathologists needed to understand the importance of the HER2 IHC evaluation. Similarly, the practise of scoring Ki67 as a global value rather than in hot spots had been recommended in the Swedish guidelines since 2022 but was still not implemented in many of the smaller laboratories. According to the recommendations of the International Ki67 in Breast Cancer Working Group (IKWG), only cases with global Ki67 ≤ 5% are considered “low” and cases with ≥ 30% are considered “high”; these cut-offs have been chosen because they ensure very low variability even between laboratories with different staining protocols [1]. Including global Ki67 in the EQA round meant that participants both had to learn the method for global scoring and, since answers were to be given as low (≤ 5%), intermediate (6–29%), or high (≥ 30%), were also made aware of the updated cut-offs.

**Fig. 1:**
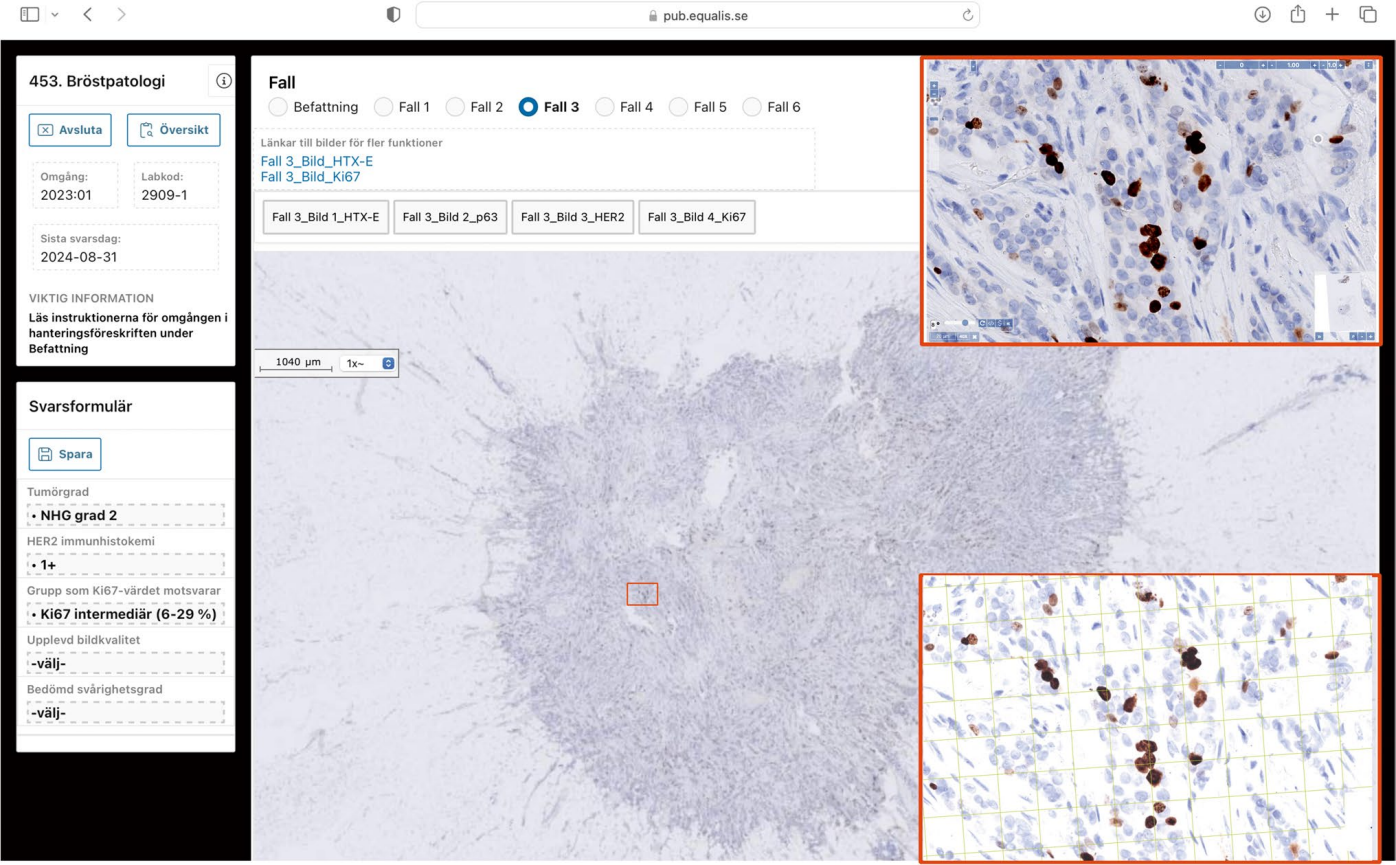
Screen-shot of Equalis pathology portal. This example shows a slide stained for Ki67. Inlaid is a magnified view of the area marked in red (top right) and the same area with the overlaid grid to facilitate scoring (bottom right, picture brightened to enhance grid in exported picture)

Figure 1 shows examples of screenshots from the Equalis Online portal for the 2023 round. To facilitate Ki67 scoring, each case contained a link to a platform (Collective Mind Research with the image viewer from Pathomation integrated), where participants could add a virtual grid, with a square size of their own choosing, on top of the slide.

Table 2 summarizes the features of the cases chosen for the 2023 round.

**Table 2:** Expected answers (accepted answers) for cases in the 2023 round

	Nottingham grade	Ki67	HER2
Case 1	1	Low (Intermediary)	2 + (1 +)
Case 2	3	High	0
Case 3	2	Intermediary	0 (1 +)
Case 4	2 (1)	Intermediary	1 +
Case 5	2	High (Intermediary)	1 + (2 +)
Case 6	3 (2)	Intermediary (High)	1 + (2 +)

*HER2* human epidermal growth factor receptor 2. Each cell shows the expected answer and (acceptable answer), if applicable

### Statistics

Interobserver reliability was calculated based on department answers, using Fleiss multi-rater kappa. The kappa values were interpreted as follows: ≤ 0.20 = none/slight; 0.21–0.40 = fair; 0.41–0.60 = moderate; 0.61–0.80 = substantial; 0.81–1.00 = excellent [2]. All calculations were performed in SPSS v.28 (IBM, Armonk, USA).

## Results

### The 2021 round

**Fig. 2:**
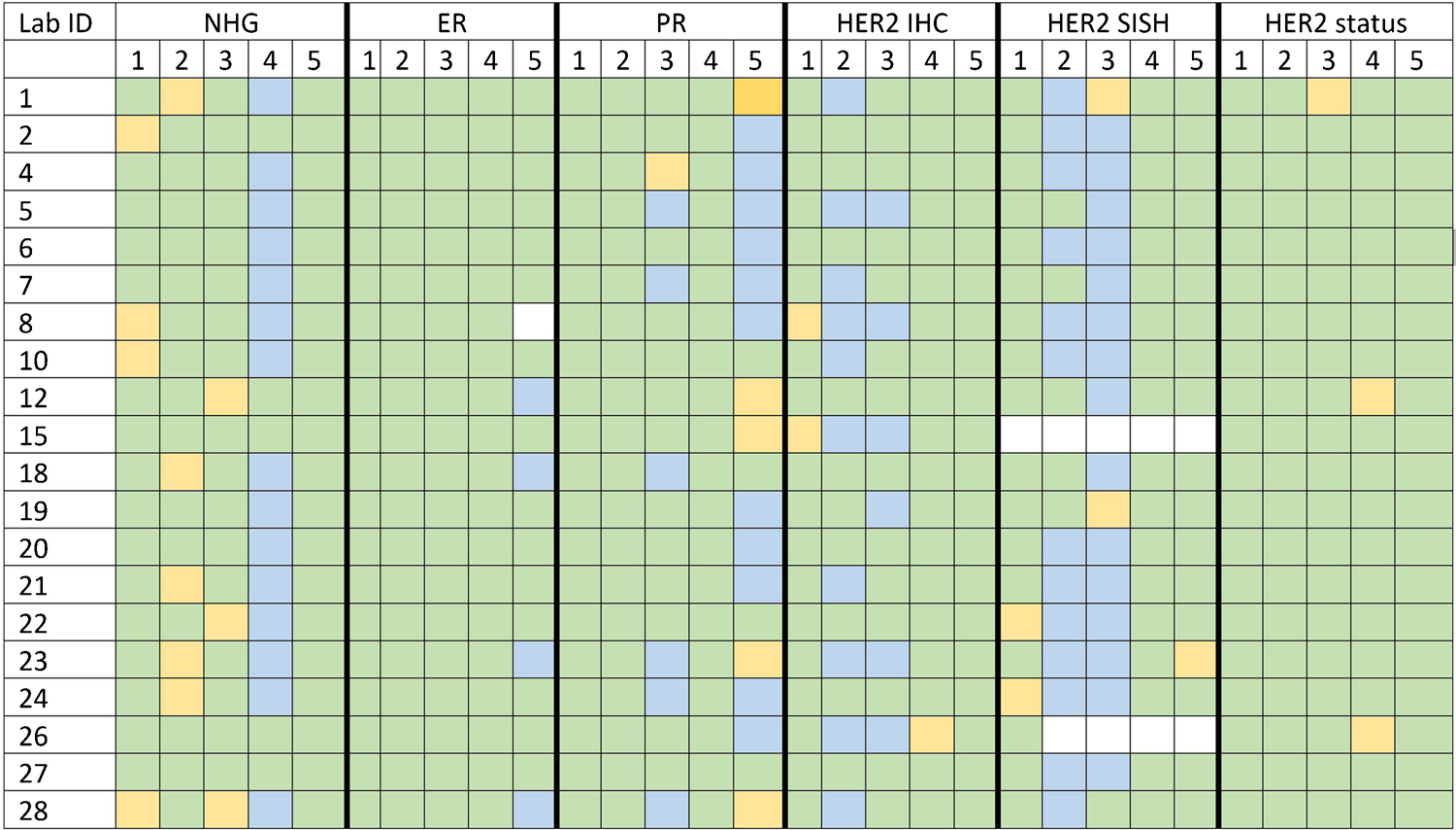
Results of the 2021 round. Green box, expected answer; blue box, accepted answer; yellow box, incorrect answer; white box, no answer registered

Twenty-four laboratories participated, of which three were Norwegian and twenty-one were Swedish. Four labs (one Norwegian) submitted only individual answers and thus were not eligible for comparison between laboratories. The results for the answers submitted as department answers are summarized in Fig. 2.

### Nottingham grade

For all five cases together, the proportion of correct answers (expected or accepted) was 88%. Generalized kappa value was moderate, 0.50 (95% confidence interval (CI) 0.45–0.55) with the highest agreement (0.70; 95% CI 0.63–0.76) for NHG 3, lowest for NHG1 (0.13; 95% CI 0.07–0.20), and NHG2 in between (0.42; 95% CI 0.36–0.49). There was only one NHG1 case, which also had NHG2 as accepted answer.

### ER and PR

All submitted answers for ER were either expected (96%) or accepted (4%), meaning that agreement was excellent (kappa = 1) if considering only the categories negative (< 10%) and positive (≥ 10%). Agreement using the three categories of 0%, 1–9%, and ≥ 10% was still substantial, with kappa 0.79 (95% CI 0.73–0.85). For PR, there were six (6%) incorrect answers; five of these were for case 6, which was an unusual ER-negative but PR-positive tumour. Kappa value for positive (≥ 10%) vs negative (< 10%) PR was 0.75 (95% CI 0.69–0.82). Using a 20% cutoff to separate low PR vs high PR resulted in slightly lower agreement (kappa 0.69; 95% CI 0.63–0.78).

### HER2

The immunohistochemistry scores for HER2 were correct or acceptable for 97% of submitted answers with a moderate agreement between categories, kappa 0.54 (95% CI 0.50–0.60). Agreement was excellent for HER2 3 + (0.94; 95% CI 0.88–1.00), moderate for 2 + (0.47; 95% CI 0.41–0.54), and only fair for 1 + (0.22; 95% CI 0.15–0.28). There were no true 1 + cases in the round, but two of the 2 + cases had 1 + as acceptable answer.

Final HER2 status (amplified/non-amplified) was correct in 97% of answers, while the SISH evaluations were more divergent with five incorrect answers (5%) and 28 acceptable (31%). These related mainly to cases 2 and 3 where the expected answers were Group 2 and Group 4, respectively. The majority of participants labelled these as Group 5.

#### The 2023 round

**Fig. 3:**
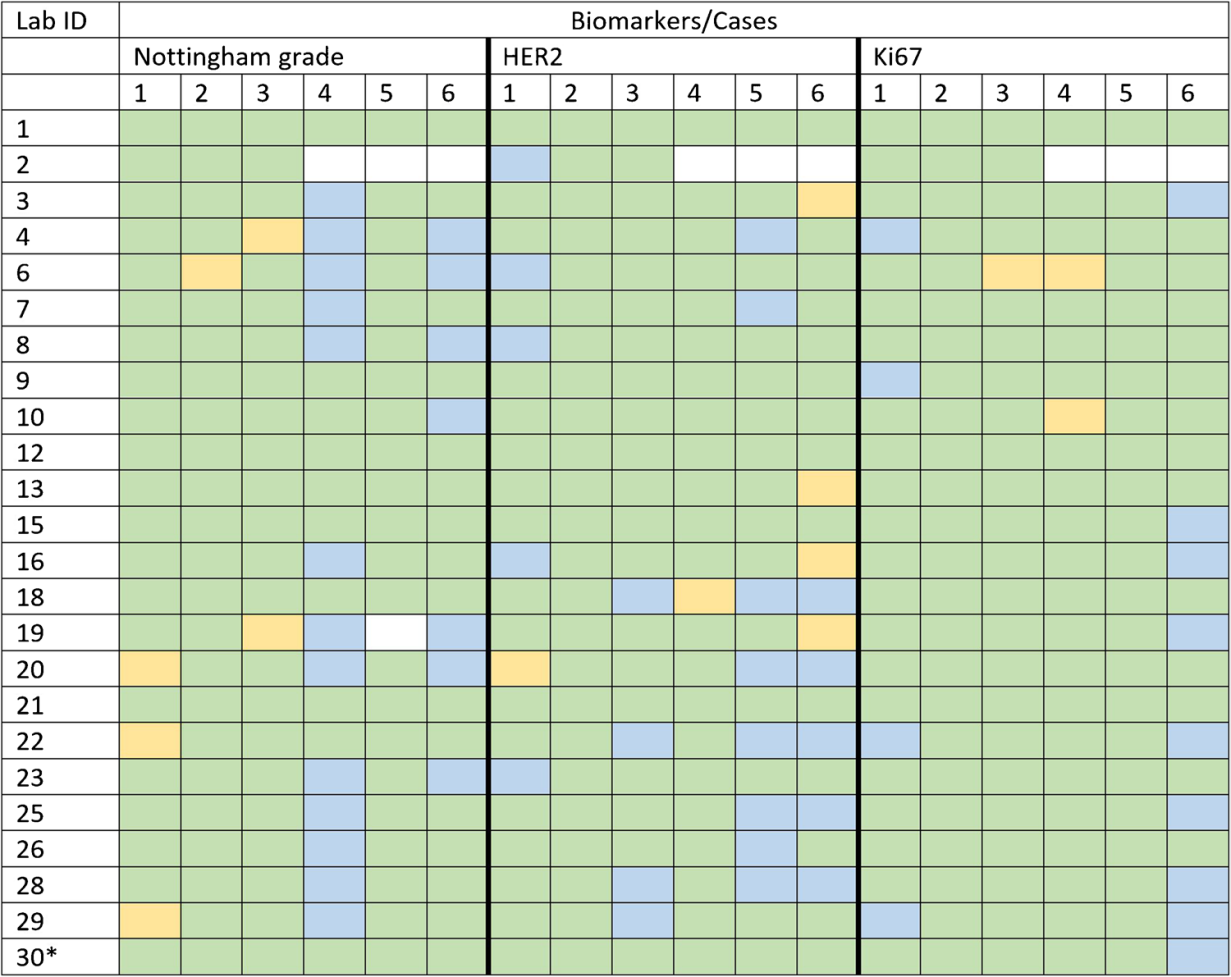
Results of the 2023 round. Green box, expected answer; blue box, accepted answer; yellow box, incorrect answer; white box, no answer registered

Twenty-five pathology laboratories participated (whereof three from Norway), and only one laboratory chose to provide individual answers only. The results for the answers submitted as department answers are summarized in Fig. 3.

### Nottingham grade

For all six cases together, the proportion of correct answers (expected or accepted) was 96%. Generalized kappa value was substantial, 0.64 (95% CI 0.60–0.68) with the highest agreement (0.79; 95% CI 0.74–0.84) for NHG 3, lowest for NHG2 (0.55; 95% CI 0.49–0.60), and NHG1 in between (0.61; 95% CI 0.56–0.66).

### HER2

The proportion of correct answers was 96% also for HER2 with a moderate interobserver agreement of 0.54 (95% CI 0.50–0.57). While agreement was substantial for the category of HER2 0 (0.78; 95% CI 0.72–0.82), it was fair (0.40; 95% CI 0.35–0.45) to moderate (0.44; 95% CI 0.38–0.49) for categories 2 + and 1 + respectively; there were no HER2 3 + cases in this round.

### Ki67

The highest proportion of correct answers was achieved for Ki67, with 98%. Overall agreement was substantial (0.61; 95% CI 0.57–0.65) but somewhat lower for the intermediary group (0.53; 95% CI 0.48–0.58). For high and low groups, the kappa values were 0.65 (95% CI 0.60–0.71) and 0.69 (95% CI 0.64–0.74), respectively.

## Discussion

### Grade

The Nottingham grading system for breast cancer has been around for more than 30 years [3] and is widely recognized as a significant prognostic factor. The main problem with this grading system, as with all three-tiered systems, is that the middle grade—NHG2—does not really provide the oncologist much aid as to whether the patient would benefit from adjuvant chemotherapy or not. Interobserver agreement in grading is also known to be less than ideal [4] with lower agreement for NHG2 than for NHG1 or NHG3 [5] and the agreement may be worse working with digital slides than glass slides [6]. The results of both the 2021 and the 2023 EQA rounds indicate that pathologists are good at identifying the high-grade tumours but less certain about separating the NHG1 from the NHG2. To ensure the best treatment for patients in an era where all Swedish pathology laboratories are digitized, then it is vital that pathologists regularly calibrate their grading in a digital format and not on glass slides. There are already AI-based digital image analyses dividing the NHG2 group into “NHG1-like” and “NHG-3-like” categories with significant prognostic impact [7, 8]. It is likely that there will soon be algorithms assisting with the entire grading, but the need for human supervision and final diagnosis will remain unchanged for the foreseeable future.

### Ki67

Perhaps no biomarker in breast cancer has been debated as much as Ki67, which has its own dedicated international working group working on clinical implementation since 2010 [9]. Every pathologist is aware of the problem of trying to get any two colleagues to agree on what is brown and what is blue when counting nuclei. A study based on the Swedish breast cancer register identified Ki67 as the parameter with the highest inter- and intra-laboratory variation, when comparing it to ER, PR, NHG, and HER2 [10]. This is troublesome, given the importance of Ki67, and how well it correlates with eg the Prosigna Risk of Recurrence Score [11]. The current recommendations of only calling tumours with a global Ki67 of ≤ 5% “low”, and only those ≥ 30% “high” are partly chosen to ensure a better concordance. In our EQA, this seems to have worked, giving Ki67 the highest overall correct answers of all three biomarkers. Most laboratories in Sweden already use AI for assistance with Ki67-scoring. Almost all EQA schemes require their samples to be treated as routine samples would be, but this is clearly not the case if we do not allow the use of different digital slide formats and the AI tools that the pathologists routinely use. Future EQAs must inevitably transition into distributing digital slides that may be uploaded onto the laboratory’s usual viewer.

### HER2

The problems for HER2 are similar to Ki67: to get pathologists to agree on whether something is “weak” brown staining or “moderate” brown staining is a daunting task. With one exception in each round, it was encouraging that no other laboratories misinterpreted 2 + cases as 3 + , as this would have serious implications for the patient. At least one of these incorrect answers were submitted by a laboratory with no in-house ISH testing, giving the pathologists less clinical experience with outcome in borderline HER2 tumours. The final HER2 assessment in 2021 (pre HER2-low era) was correct in 97% of cases, and one of the three incorrect answers may have been unintentional as the laboratory answered IHC 2 + and Group 5, but the final status as HER2-positive; this combination otherwise answered as negative by the same lab. Registry data also supports that there is no statistically significant variation in proportion of HER2 positive (3 + and/or amplified) tumours between laboratories [10]. In the 2023 round, however, one of the goals was to draw attention to the HER2-low category, and borderline cases were intentionally selected, which was reflected in the lower kappa value this year compared to 2021. As the same antibody and protocols were used for all cases in 2021 as well as 2023 (4B5, Roche/Ventana), this should not have contributed to the variability.

### ER/PR

The hormone receptors (HR), ER and PR, are important prognostic and predictive factors in breast cancer [12]. In accordance with previous registry-based findings [10], we demonstrated an outstanding agreement among pathology laboratories to detect ER-positive and negative cases. There is an ongoing debate whether ER low breast cancer patients should be treated as TNBC patients [13, 14] or if endocrine therapy is still the standard of care in this patient group [15] which makes the pathologist’s assessment critical in the lower end of ER dynamic range. The agreement between participating laboratories was still substantial when ER low (1–9%) was added as a separate category in the comparison. According to international recommendations, PR can be used to aid in differentiating between Luminal A-like and Luminal B-like tumours when no gene expression analysis is available [16]. Here we found a good agreement between pathology laboratories in distinguishing PR low and PR high cases.

### What makes this Swedish EQA scheme unique?

Morphology is still the golden standard in cancer diagnosis. The Equalis EQA schemes in pathology focus on morphological assessment in digitized routine slides as well as immunohistochemical assessment of the same case. As already mentioned, most EQA providers lack schemes for routine histomorphology, focusing instead on immunohistochemistry and genetics. Furthermore, there are very few EQA schemes that allow digital evaluation of clinical biomarkers. The College of American Pathologists (CAP) provide a Performance Improvement Program in Surgical Pathology (PIP) but the program consists of neoplastic and non-neoplastic lesions from any organ. It is certainly a valuable educational tool but not sufficient for a laboratory that wants to validate its diagnostic process in any specific field. The Swedish EQA schemes do not aim to compete with the large international EQAs such as NordiQC, UKNEQAS, QuIP, or CAP [17–20]—they are indispensable tools for ensuring consistency in staining and interpretation of immunohistochemical slides—but instead complement them.

Compared to other established EQA programmes like CAP, NordiQC, and UK NEQAS, the Swedish scheme’s bottom-up approach offers unique flexibility and adaptability to local needs. For instance, while CAP’s EQA programmes provide extensive proficiency testing across multiple disciplines with a strong focus on educational insights and benchmarking against large peer groups, the Swedish programme emphasizes regional specificity and the inclusion of morphology-based assessments, which are often overlooked in larger schemes. Similarly, NordiQC focuses on immunohistochemical quality control with detailed protocol assessments and critical assay performance controls, but it may not address the local variations and specific challenges highlighted by the Swedish programme. UK NEQAS offers a broad range of EQA services, yet the Swedish scheme’s rapid feedback loops ensure continuous quality improvement tailored to the evolving clinical landscape. For instance, in the latest round of the Swedish EQA program in 2023, HER2-low was in focus as there is urgent need to address the potential variability due to the new clinical therapies and the large international programmes are yet to cover the new analytical aspect related to this new therapy. Furthermore, unlike with international EQAs, the results of the Swedish Digital EQA scheme are directly linked with the national pathology guidelines. For example, the agreement between pathologists performing grade was moderate and comparable with that of the UK NEQAS results in 2021 [21]. Due to this, more focus and details have been put on NHG in the national recommendations. In the next, follow-up round of 2023, we found and improved agreement among participating pathologists performing NHG.

In conclusion, the Equalis Swedish Pathology Digital External Quality Assessment scheme is a bottom-up initiative empowering local pathology communities to contribute to the improvement of processes, fostering a culture of continuous quality improvement and education achieving the state-of-the-art pathology. It is a complement to the large international EQA schemes and allows for the identification and resolution of problems at the local level, which may be overlooked in a top-down approach. Lastly, this bottom-up EQA approach can highlight the most problematic variables among the rapidly changing prognostic and predictive biomarkers in cancer therapy.

## Data Availability

The data are not publicly available due to restrictions by Swedish and European law. Data are available for researchers with relevant ethical approvals to access upon request.
